# Bridging Generations: Brain Health Education with Older Adults and Undergraduates in Community Settings

**DOI:** 10.1093/geroni/igaf122.3723

**Published:** 2025-12-31

**Authors:** Jian Han, Morgyn Edwards, Madison Edwards, Khalil McKoy, Ashley Sanderlin, Travonia Hughes

**Affiliations:** North Carolina Agricultural and Technical State University, Greensboro, North Carolina, United States; North Carolina Agricultural and Technical State University, Greensboro, North Carolina, United States; North Carolina Agricultural and Technical State University, Greensboro, North Carolina, United States; North Carolina Agricultural and Technical State University, Greensboro, North Carolina, United States; North Carolina Agricultural and Technical State University, Greensboro, North Carolina, United States; North Carolina Agricultural and Technical State University, Greensboro, North Carolina, United States

## Abstract

Intergenerational service learning has the potential to bridge the gap between younger and older populations while promoting mutual educational and social benefits. This project examined the outcomes of a community-based educational intervention in which undergraduate biology students delivered health-related presentations to older adults residing in independent living facilities across North Carolina. Eleven students developed and delivered 10 interactive presentations on brain health, nutrition, physical activity, and healthy aging. Sessions also featured hands-on activities such as pill sorting games, exercise bingo, and MyPlate-based nutrition planning to enhance participant engagement. Quantitative data from pre- and post-program surveys (analyzed using paired t-tests at p = 0.05) revealed significant student learning gains in communication, nutrition, and activity planning. The Classroom Undergraduate Research Experience (CURE) survey further supported these findings, with strong gains noted in the “Understanding” domain of Bloom’s taxonomy. Older adult participants rated the sessions highly in satisfaction (79%) and engagement (76%), citing both the educational value and interactive components. Findings suggest that intergenerational service learning effectively promotes healthy aging while enhancing students’ academic skills, empathy, and commitment to community engagement.

